# Betulinic Acid Modulates the Expression of HSPA and Activates Apoptosis in Two Cell Lines of Human Colorectal Cancer

**DOI:** 10.3390/molecules26216377

**Published:** 2021-10-22

**Authors:** Laphatrada Yurasakpong, Chanin Nantasenamat, Saksit Nobsathian, Kulathida Chaithirayanon, Somjai Apisawetakan

**Affiliations:** 1 Department of Anatomy, Faculty of Science, Mahidol University, Rama VI Road, Ratchathewi, Bangkok 10400, Thailand; laphatrada.yru@gmail.com (L.Y.); kulathida.cha@mahidol.ac.th (K.C.); 2 Center of Data Mining and Biomedical Informatics, Faculty of Medical Technology, Mahidol University, Bangkok 10700, Thailand; chanin.nan@mahidol.edu; 3 Nakornsawan Campus, Mahidol University, Nakonsawan 60130, Thailand; saksit.nob@mahidol.ac.th; 4 Department of Anatomy, Faculty of Medicine, Srinakharinwirot University, Wattana, Bangkok 10110, Thailand

**Keywords:** betulinic acid, HSPA, apoptosis, colorectal cancer

## Abstract

Betulinic acid (BA) is a pentacyclic triterpene usually isolated from botanical sources. Numerous studies have reported the inhibitory effect of BA against human colorectal cancer cells (CRC). However, its effect on the expression of the molecular chaperone HSPA is unclear. The aim of this research is to investigate the anti-cancer activities of BA purified from *Piper retrofractum* and study its effect on the expression of HSPA in colorectal cancer HCT116 and SW480 cells. The viability of both cancer cells was reduced after they were treated with an increasing dosage of BA. Flow cytometry assay revealed that levels of cell apoptosis significantly increased after incubation with BA in both cancer cells. Pro-apoptotic markers including Bax, cleaved-caspase-3 and cleaved-caspase-9 were increased while anti-apoptotic marker Bcl-2 was decreased after BA treatment. Western blot also showed that the expression of HSPA fluctuated upon BA treatment, whereby HSPA was increased at lower BA concentrations while at higher BA concentrations HSPA expression was decreased. Preliminary molecular docking assay showed that BA can bind to the nucleotide binding domain of the HSP70 at its ADP-bound state of the HSP70. Although further research is needed to comprehend the BA-HSPA interaction, our findings indicate that BA can be considered as potential candidate for the development of new treatment for colorectal cancer.

## 1. Introduction

Human heat shock proteins (HSPs) are classified into two classes according to their activity and relative molecular weight [1]. Low molecular weight HSPs are ATP-independent and have a molecular weight of 34 kDa or less. High molecular weight HSPs, or ATP-dependent HSPs, consist of HSP110, HSPC (HSP90), HSPA (HSP70) and HSP60 [2]. HSPA1, or also known as HSP70 [3], is a chaperone that functions in protein folding, refolding and transport [4], and is also a major stress inducible isoform of HSPA. HSPA proteins are composed of two domains including an N-terminal ATPase domain (NBD) (~44-kDa) with ATPase activity and a C-terminal substrate binding domain (SBD) (~25-kDa) that binds polypeptide substrates. Previous studies demonstrated that the increased expression of HSPA in various human cancer cells was associated with resistance to treatment due to suppression of apoptosis [5,6], while the suppression of HSPA expression can enhance drug sensitivity [7]. The expression of HSPA is a potential biomarker for human colorectal carcinoma (CRC) because HSPA is abundantly expressed in serum and cancer tissue, and was found to correlate with cancer stage and the patient’s survival rate [8,9]. Therefore, the oncogenic property of HSPA made it a potential target in cancer therapeutics.

Currently, the HSPA inhibitors in CRC treatment include adenosine-derived HSPA inhibitors, dihydropyrimidine-derived HSPA inhibitors, GRP78 inhibitors, rhodocyanine-derived HSPA inhibitors, imidazole-derived HSPA inhibitors and phenylethylsulfonamide-derived HSPA inhibitors [10]. In addition, natural compounds with HSPA inhibitory activity were investigated in various cancer cell lines. Quercetin is a flavonoid, which was found to suppress cancer cell proliferation via the inhibition of HSF1, an upstream regulator of HSPA [11]. The modified encapsulated form of quercetin (Q-PEGL) can also inhibit the expression of HSPA, leading to the induction of apoptosis in CRC tissues [12]. Kahweol is a diterpenoid with the ability to promote apoptosis of CRC cancer cells via the downregulation of the HSPA [13]. Cantharidin can inhibit transcriptional activity of HSF1, an upstream regulator of HSPA, leading to a reduction in the expression Bcl-2, Bcl-xL and Mcl-1 and the induction of apoptosis [14]. Further research is needed to better understand the interaction of these naturally derived inhibitors with HSPA and evaluate their potential use as a CRC treatment drug.

**Figure 1:**
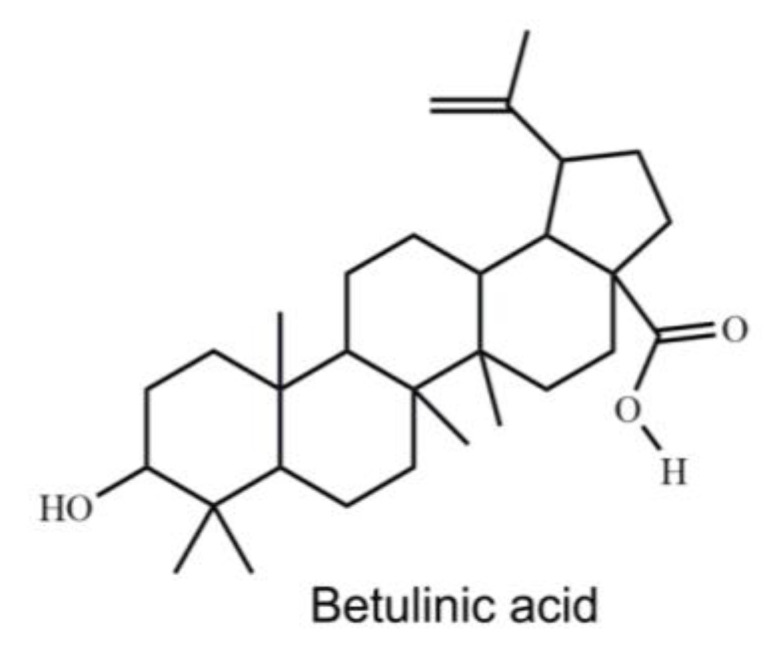
The chemical structure of BA.

Pentacyclic triterpenes are natural substances which are abundantly found in a wide variety of plants [15]. The anti-cancer of these triterpenes and their derivatives has been extensively investigated especially on apoptosis and angiogenesis including maslinic acid [16], oleanolic acid [17], ursolic [18] and also betulinic acid (BA) (Figure 1). BA is a pentacyclic triterpenoid usually isolated from plants [19,20]. In CRC cells, BA was found to increase the production of the reactive oxygen species (ROS), Bax and cleaved caspase-3, leading to mitochondrial apoptosis in the HCT116 cell line [21]. However, the effect of BA on the expression of HSPA, a hallmark protein in CRC, is yet to be investigated. In the present study, we aim to investigate the effect of BA treatment on the expression of HSPA and CRC cell viability in vitro. Our results demonstrated BA could modulate the HSPA expression, promote apoptosis in vitro, and could be used as a potential therapeutic agent to prevent the progression of CRC.

## 2. Results

### 2.1. Effect of BA on Human Colorectal Carcinoma Cell Viability

**Figure 2:**
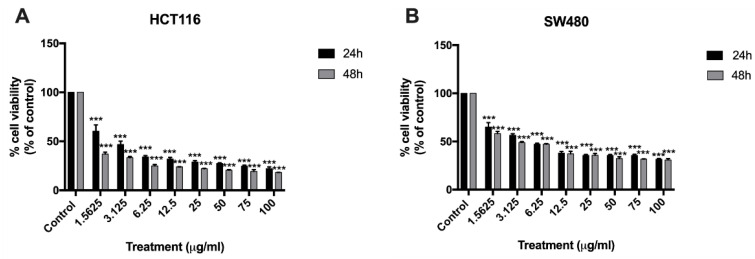
MTT assay showing the cytotoxic effect of BA at 24 and 48 h against HCT116 (**A**) and SW480 (**B**) colorectal cancer cells at BA concentrations ranging from 1.5625 to 100 ug/mL. Results are presented as mean ± standard error of the mean (SEM). Experiments were carried out in triplicate. One-way ANOVA and with Dunnett’s post hoc test were used to test statistical significance with *** indicating *p* < 0.001 vs. control group.

To determine the direct cytotoxicity of BA on human CRC cell lines, HCT116 and SW480 cells were seeded onto 96-well plates and treated with different concentrations of BA at 1.5625, 3.125, 6.25, 12.5, 25, 50, 75, 100 µg/mL for 24 and 48 h. MTT colorimetric assay was performed to evaluate the cellular viability and proliferation. The relative cell viability was calculated as a percentage relative to the untreated control cells. It was found that BA had a concentration-dependent cytotoxicity effect on both HCT116 and SW480 human colorectal cancer cells in comparison to the control (Figure 2). The half maximal inhibitory concentration values (IC_50_) of BA at 24 and 48 h were approximately 8.263 µg/mL (18.1 µM) and 6.441 µg/mL (14.15 µM) for SW480 cells (Figure 2B); and 4.11 µg/mL (9.032 µM) and 1.67 µg/mL (3.670 µM) for HCT116 cells (Figure 2A). These data indicated that treatment with BA provided significant inhibitory effect on HCT116 and SW480 cells in time- and dose-dependent manners.

### 2.2. Morphological Changes in Cells and Nucleus Are Affected by BA

**Figure 3:**
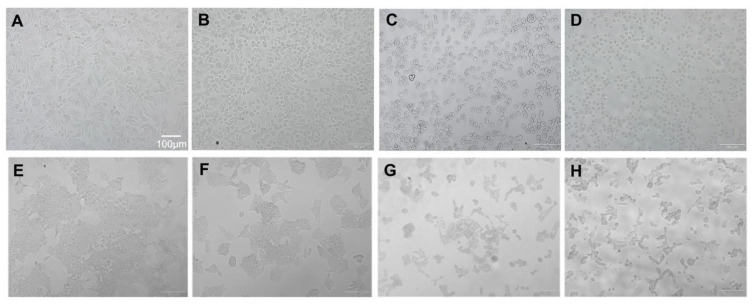
Morphological changes to HCT116 colorectal cancer cells (**A–D**) at 0, (**A**) 1.25, (**B**) 5, (**C**) and 10 µM (**D**) and SW480 colorectal cancer cells (**E–G**) at 0, (**E**) 2.3, (**F**) 5, (**G**) 10 (**H**) µM 24 h following the BA treatment.

**Figure 4:**
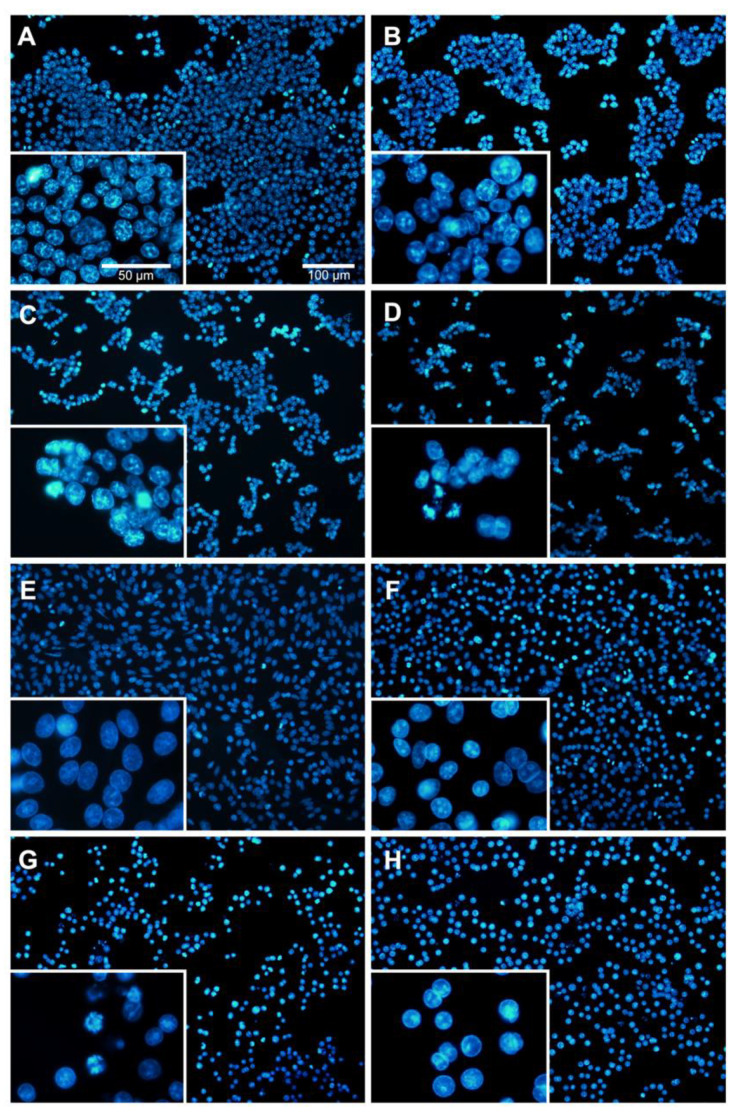
Nuclear morphology of BA-treated HCT116 (**A–D**) and SW480 (**E–G**) human colorectal cancer cells at 24 h using Hoechst staining. Different concentrations of BA were used, including 0, (**A**) 1.25, (**B**) 2.5, (**C**) and 5 µM (**D**) for HCT116; and 0, (**E**) 2.5, (**F**) 5 (**G**) and 10 µM (**H**) for SW480 cells. Cells were observed under 100 X and 50 X magnification using a fluorescence microscope.

To investigate whether BA induces apoptosis of CRC cells, morphological and nuclear changes in HCT116 and SW480 cells were studied at concentrations lower than the IC_50_ (i.e., 0, 1.25, 2.5 and 5 µM use for HCT116; and 0, 2.5, 5 and 10 µM use for SW480). As evident from Figure 3, the morphology of both cancer cells was changed including rounding up of the cell, shrinkage of cellular and nuclear volume, and the lack of intercellular contact (Figure 3B–D for HCT116; Figure 3F–H for SW480) compared to the controls (Figure 3A for HCT116; Figure 3E for SW480). As shown in Figure 4, Hoechst 33342 staining in both CRC cells after BA treatment showed more prominent nuclei with distinct apoptotic changes including nuclear fragmentation and condensation (Figure 3B–D for HCT116; Figure 3F–H for SW480) compared to the controls (Figure 3A for HCT116; Figure 3E for SW480).

### 2.3. Induction of Apoptotic Cells in Human Colorectal Cancer Cells by BA

**Figure 5:**
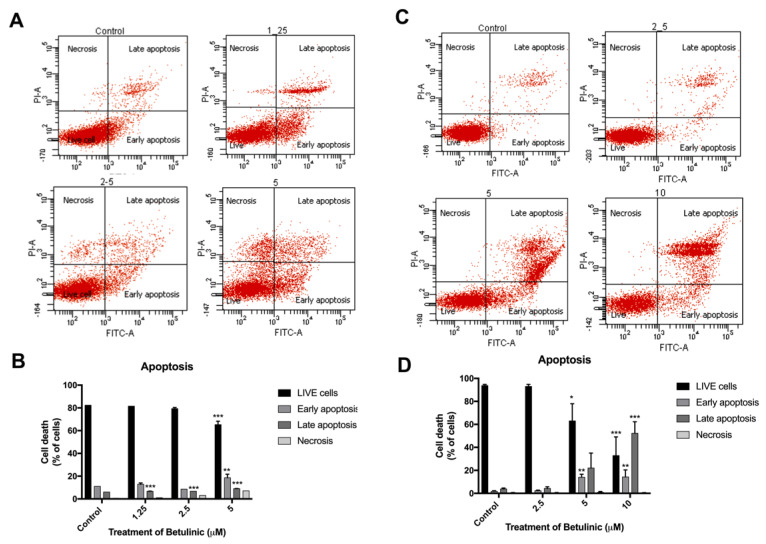
Annexin V-FITC/PI staining showing BA-induced apoptosis in HCT116 (**A,B**) and SW480 (**C,D**) human colorectal cancer cells at 24 h. Apoptotic cells are indicated by the red dots in upper and lower right quadrants of each diagram, whereby lower and upper panels represent early and late stages of apoptosis, respectively. Experiments were carried out in triplicate. One-way ANOVA and with Dunnett’s post hoc test were used to test statistical significance with * indicating *p* < 0.05, ** *p* < 0.01 and *** *p* < 0.001 vs. control group.

To study the BA-mediated cell death of HCT116 and SW480 cells, flow cytometry analysis was performed using Annexin V-FITC/PI double staining assay (Figure 5). The percentage of apoptotic cells was significantly increased in HCT116 cells after 24 h treatment with BA. Early apoptotic cells were increased to 12.9, 8.6 and 18.6%; and late apoptotic cells increased to 6.7, 6.8 and 9.05%, at 1.25, 2.5 and 5 µM, respectively. (Figure 5A,B). Likewise, in SW480 cells the percentages of the early apoptotic was increased to 2.25, 14.1 and 14.3% of cells in early apoptosis; and to 4.25, 22.0 and 52.3% of cells in late apoptosis at 0, 2.5, 5 and 10 µM, respectively, after 24 h treatment with BA compared to the untreated cells. (Figure 5C,D). These results indicated that the inhibitory effect of BA on human colorectal cancer cells, HCT116 and SW480, were due to the induction of apoptosis.

### 2.4. The Protein Expression Level of Apoptotic Markers Is Affected by BA

**Figure 6:**
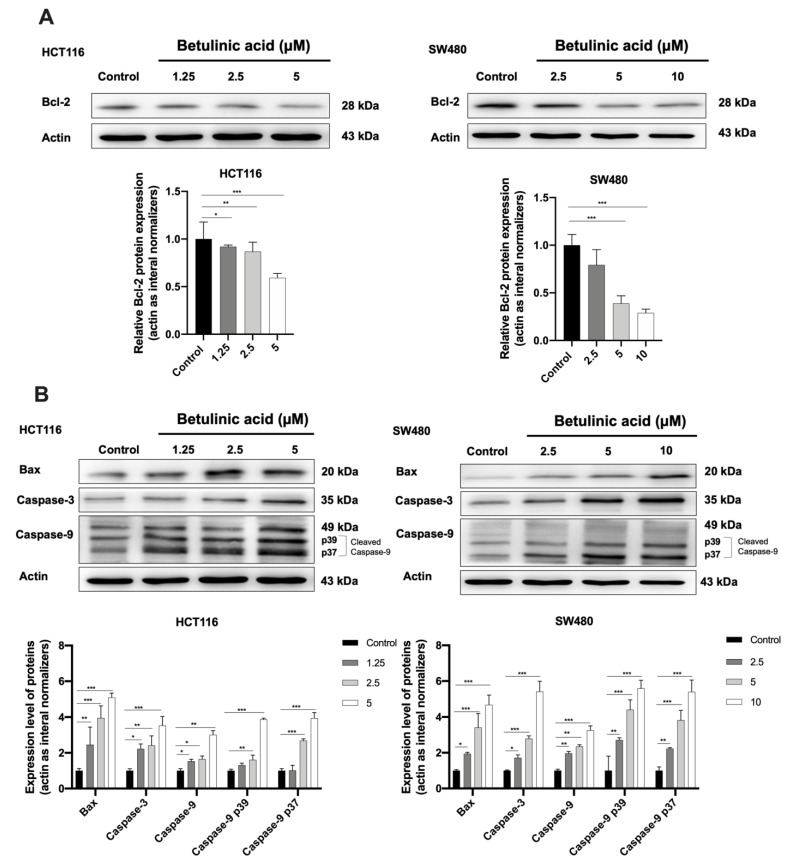
Western blot analysis showing the expression of anti-apoptotic (**A**) and proapoptotic proteins (**B**) in HCT116 and SW480 colorectal cancer cells at 24 h following the BA treatment. Actin was used as the internal control. Experiments were carried out in triplicate. Results are represented as mean ± standard error of the mean (SEM). One-way ANOVA and with Dunnett’s post hoc test were used to test statistical significance with * indicating *p* < 0.05, ** *p* < 0.01 and *** *p* < 0.001 vs. control group.

Western blot analysis was carried out to confirm the expression of BA-induced apoptosis on human colorectal cancer cells (Figure 6). Expression levels of Bcl-2, Bax, caspase-3, and cleaved caspase-9, were elucidated using western blot analysis after treatment of BA at concentrations of 0, 1.25, 2.5 and 5 µM for HCT116 (Figure 6A); and 0, 2.5, 5, 10 µM for SW480 (Figure 6B) for 24 h. It was found that both CRC cells treated with various concentrations of BA for 24 h resulted in a dose-dependent increase in apoptotic cells in comparison to the respective control group.

As presented in Figure 5, a concentration-dependent decrease in the Bcl-2 expression and an increase in Bax expression were observed in HCT116 and SW480 cells treated with BA. In addition, caspase-3 and caspase-9 were remarkably increased by BA treatment, especially at high doses (Figure 5A,B). Collectively, these results indicated that BA promoted cell death in human CRC cells via the induction of intrinsic apoptosis.

### 2.5. HSPA Expression Is Regulated by BA

**Figure 7:**
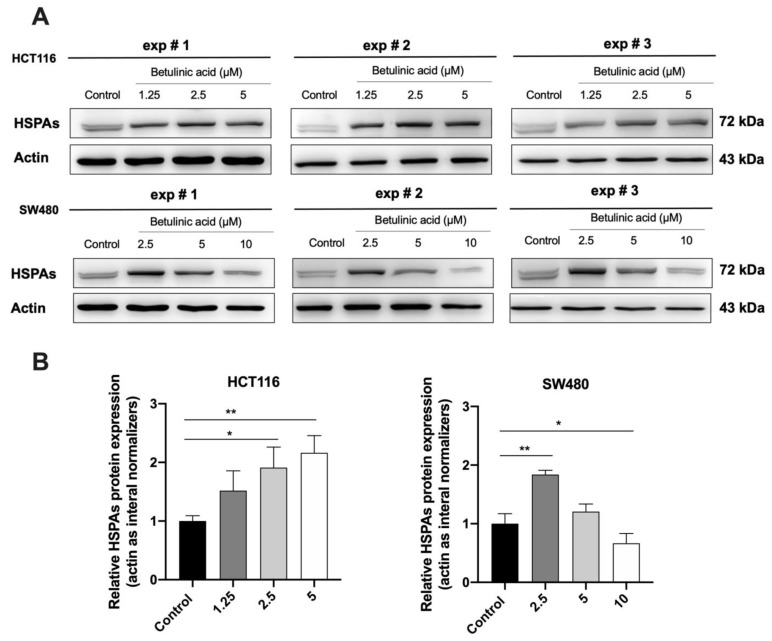
Western blot analysis showing the HSPA’s expressions following the BA treatment in HCT116 and SW480 cell lines (**A**) and their corresponding quantifications (**B**). Actin was used as the internal control. All experiments were triplicate and the results were represented as mean ± standard error of the mean (SEM). One-way ANOVA and with Dunnett’s post hoc test were used to test statistical significance with * indicating *p* < 0.05, and ** *p* < 0.01 vs. control group.

Western blot and molecular docking assay were performed to study the relationship between BA and HSPA. Western blot analysis further confirmed that the 70-kD was in fact the HSPA (Figure 7A). The expression level of HSPA was increased after treatment with the BA at 1.25, 2.5 and 5 µM in both of CRC, while the HSPA level was significantly reduced to about 0.7-fold at 10 µM of BA treatment in SW480 cells, compared to the control group.

**Figure 8:**
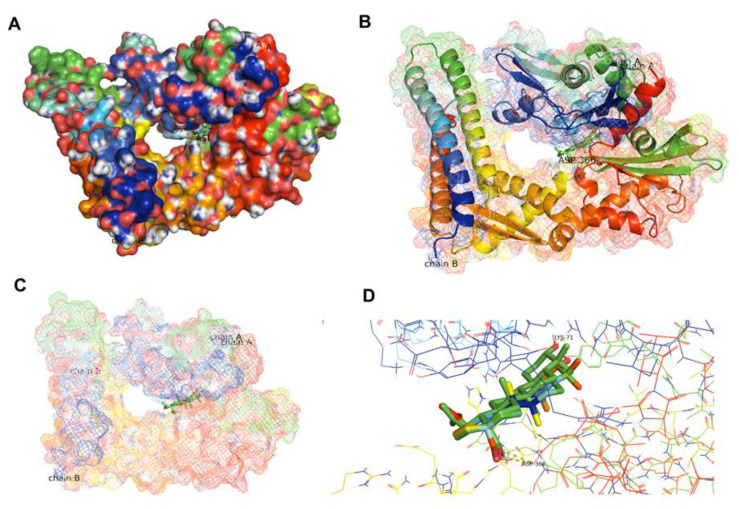
Docked structures of HSP70′s NBD in ADP-bound state (PDB ID 5AQF) (**A,B**) indicating the binding of BA to the ADP-binding region of HSP70 (**C**) with a close-up view (**D**).

**Figure 9:**
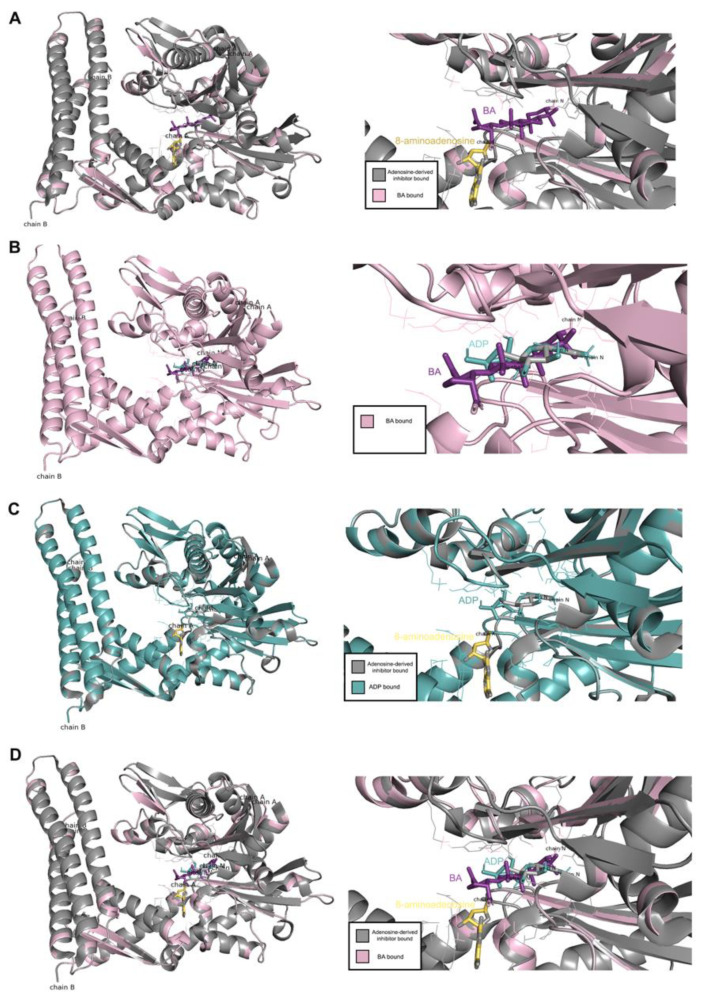
Comparison between the docking of BA and adenosine-derived inhibitor, (**A**) BA and ADP-bound structure, (**B**) adenosine-derived inhibitor and ADP-bound structure (**C**) as well as BA and adenosine-derived inhibitor and ADP-bound structure (**D**).

To confirm the interaction between BA and the HSPA protein, molecular docking was performed. In the present study, *Homo sapiens sp* HSP70 (PDB ID 5AQF) was used. Result showed that BA could bind to the nucleotide binding domain of the HSP70 at the ADP-bound state of the HSP70 chaperone cycle. The carbonyl group (COOH group) of BA could protrude into the polar pocket to form hydrogen bonds with the side chain of Asp366 and Lys71. The binding affinity between HSP70 and BA was −7.4 kcal/mol (Figure 8). In comparison to 8-aminoadenosine (i.e., an adenosine-derived inhibitor), it was found that BA was bound at a different region of HSP70 (Figure 9). Superimposition was performed to examine and compare the conformational changes to the HSP70 upon binding BA and/or 8-aminoadenosine in the presence or absence of the ADP-bound structure. Results indicated that there were conformational changes to HSP70 after docking to BA. Superimposition with the docked structures of HSP70 and 8-aminoadenosin revealed that there was an incomplete overlapping, thereby implying that the conformational change caused by BA was different from that of 8-aminoadenosine (Figure 9A), similar to when the superimposed 8-aminoadenosine was docked to the ADP-bound structure (Figure 9C). Conversely, comparison of the BA-HSP70 docked structure to that of the HSP70-ADP docked structure indicated a complete overlap (Figure 9B). This indicates that BA might bind to HSP70 in a similar fashion to that of ADP to the HSP70.

## 3. Discussion

Terpenes, namely terpenoids or isoprenoids, are organic composites usually isolated from botanical sources [22]. Triterpenes (triterpenoids) consist of 30 carbon terpenoids, and are present in different plant organs including roots, leaves, seeds and bark. Anti-cancer properties of natural triterpenoids have widely been investigated especially on apoptosis and angiogenesis [15]. Maslinic acid was found to induce apoptosis of HT29 colon cancer cells by activating mitochondrial apoptotic pathway [16]. Similarly, uvaol was recently found to increase the expression of HSP-60, downregulate the AKT/PI3K pathway and activate mitochondrial apoptotic pathway in HepG2 hepatocellular carcinoma cell line [23]. Oleanolic acid was able to induce cell cycle arrest and induce apoptosis in B16-F1 melanoma cells [17]. BA, similar to other triterpenoids, has been thoroughly investigated as a potential therapeutic in many human cancers, including melanoma carcinoma [24], breast carcinoma [25], prostate carcinoma [26], lung carcinoma [27], glioblastoma [28] and colorectal carcinoma [21].

Our report is the first study to report the inhibitory action of BA against colorectal cancer cells through the inhibition of HSPA, which was accompanied by the induction of apoptosis of CRC cancer cells. We first conducted an MTT cell viability assay to study the cytotoxicity of BA in vitro. We found that the viability of both the CRC cell lines was inhibited in a time-and-concentration dependent manner after BA treatment. Sub-IC50 concentrations of BA were used in subsequent experiments [29]. To further investigate the nuclear morphology change, light microscopy and Hoechst 33342 nuclear staining were used. The results showed that morphological features of apoptotic cells were presented after BA treatment including cell shrinkage, fragmentation and formation of apoptotic bodies [30]. Similarly, Hoechst staining showed nuclear fragmentation, DNA condensation and chromosomal DNA fragmentation, which are indicators of apoptosis. Flow cytometry analysis was performed to further evaluate the induction of apoptotic cell death in both colorectal carcinoma cells. Early and late apoptotic cells are distinguished by the staining of Annexin V/PI, whereby the early apoptotic cells are annexin V positive but PI negative, and late apoptotic cells both annexin and PI positive [31,32]. In our experiments, both the HCT116 and SW480 cells were annexin V/PI positive, suggesting that apoptotic cell death was triggered. To explore the potential molecular mechanism of BA-induced apoptosis, western blot analysis found that BA treatment caused a significant reduction in key apoptosis-associated proteins such as pro-apoptotic Bcl-2 in HCT116 and SW480 cells, while Bax, cleaved-capsese-3 and cleaved-capsese-9 were increased. Previous studies revealed that BA had a potential to enhance the pro-apoptotic Bax and Mcl-1 levels in melanoma, neuroblastoma, glioblastoma and colorectal carcinoma cells [21,33,34,35]. However, BA affects the expression of Bcl-2 protein in a cell type-dependent manner. No change in the expression of Bcl-2 was observed in neuroblastoma and squamous cell carcinoma after BA treatment [34,35]. On the contrary, an enhanced Bcl-2 level was found in glioblastoma cells, while the expression of Bcl-2 protein was reduced in colorectal carcinoma [21,33,34,35]. The ratio of Bax/Bcl-2 regulates the cytochrome *c* release from mitochondria. During the cytochrome *c* secretion, cytochrome *c* binds to Apaf-1 and pro-caspase-9 and cleaved-caspase-9 [36,37]. The cleaved-caspase-9 then activates caspases-3 and -7. Numerous studies indicated that BA has the ability to induce apoptosis in several cancer cells, noted by the upregulation of caspase-9 and caspase-3 activities [38]. Taken together, our finding suggested that BA could induce intrinsic apoptosis pathway in both CRC cell lines.

We found that the HSPA expression was modulated by BA. HSPA plays an important role in the anti-apoptotic factor by directly or indirectly modulating the intrinsic apoptotic pathways [39]. In cancer cells, inhibition of HSPA causes the cancer cell to be vulnerable to death stimuli-induced apoptosis, while rich intracellular HSPA has long been known to inhibit apoptosis [40]. HSPA inhibits the intrinsic apoptosis pathway by preventing the translocation of Bax into the mitochondria, leading to the blockage of its downstream targets including cytochrome *c* and caspases [41,42]. We examined the expression level of HSPA following BA treatment using western blot. We found that the HSPA expression was markedly decreased in both HCT116 (at 5 µM) and SWU480 cells (at 10 µM), although there was a slight increase in HSPA expression at lower concentrations (1.25 and 2.5 µM). According to previous studies, the expression of HSPA can be induced by multiple mechanisms such as heat, glucose-regulated protein 78 (GRP78), a member of the HSPA family, and reactive oxygen species (ROS) [43,44,45]. Interestingly, the BA treatment significantly upregulated GRP78-mediated endoplasmic reticulum (ER) stress in breast cancer cells [46]. Therefore, these findings indicate that upregulation of HSPA at 1.25 and 5 µM concentrations may be involved with the enhanced ROS production. However, there is evidence that suggests otherwise. Uvaol, another pentacyclic triterpenoid, was found to upregulate HSP60 while at the same time downregulate the AKT/PI3K pathway, thus promoting apoptosis in HepG2 cells [18]. This finding indicates that upregulation HSPs in this case is not positively correlated with cancer progression or inhibition of apoptosis, meaning the upregulation of HSPA and the induction of apoptosis may be concomitant events.

While the expression of HSPA was increased at lower concentrations (1.25 and 5 µM) of BA treatment, we found that BA treatment at higher concentrations could significantly downregulate the expression of HSPA in both HCT116 (at 5 µM) and SWU480 cells (at 10 µM). Although the results are encouraging, it is unclear whether HSPA was directly or indirectly inhibited by BA. As shown in our molecular docking assay, BA may interact directly with the HSPA protein that consequently leads to the stabilization of the ADP-bound state of HSPA. This finding is similar to the effect of MKT-077, an allosteric inhibitor of HSP8A, which selectively interacts with the ADP allosteric state stalling the protein refolding cycle [47]. As a result, the MKT-077 bound HSP8A cannot liberate its substrate, and is recognized by C-terminal HSPA interacting protein (CHIP), a ubiquitin ligase that encourages clearance via the proteasome activity [48]. Similarly, avicins (triterpenoid electrophiles) can degrade HSPA by stress-induced ubiquitination which also involves proteosome activity [49]. Another possibility is that BA might inhibit the expression of HSPA indirectly. It was previously reported that similar compounds, including triptolide [50], cantharidin [13] and quercetin [10], could bind to heat shock transcription factor 1 (HSF1), a regulator of HSPA. These compounds inhibited the transcriptional activity of HSF1 by preventing it from binding to the HSPA promotor, reducing the HSPA mRNA level, thus inhibiting the HSPA protein expression.

## 4. Materials and Methods

### 4.1. Cell Lines, Reagents and Antibodies

Colorectal carcinoma cell lines, HCT116 and SW480, were purchased from the American Type Culture Collection (ATCC, Manassas, VA, USA). MTT (3-(4,5-Dimethylthizol-2-yl)-2,5-diphenyltetrazolium bromide) and dimethyl sulfoxide (DMSO) were bought from Sigma-Aldrich (St. Louis, MO, USA). Annexin V-FITC Apoptosis Detection Kit was purchased from BD Pharmingen, Inc, (San Jose, CA, USA). The culture medium, RPMI 1640 and Dulbecco’s modified Eagle’s medium (DMEM), fetal bovine serum (FBS), penicillin and streptomycin were purchased from Grand Island Biological Company (GIBCO, Grand Island, NY, USA). The antibodies were obtained from the following sources: Bax (#2772), caspase-9 (#9502), and (HSPA) HSP70 (#4872) were purchased from Cell Signaling Technology, Inc. (Danvers, MA, USA); Bcl-2 (ab196495), caspase-3 (ab90437), β-actin (ab227387), and rabbit polyclonal secondary antibody goat anti-rabbit IgG H and L (HRP) (ab97100) were from Abcam (Cambridge, MA, USA). Hoechst 33342 was procured from Thermo Fisher Scientific (Waltham, MA, USA).

### 4.2. Plant Materials

The leaves and twigs of *Piper retrofractum* were collected in May 2017 from Chanthaburi province, Thailand. A voucher specimen (BKF no. 68866) of *P. retrofractum* was deposited at the Forest Herbarium, Royal Forest Department, Bangkok 10900, Thailand.

### 4.3. Extraction and Isolation

Air-dried and finely powdered leaves and twigs (2.1 kg) were percolated by methanol (48 L × 5 days × 5 times) at room temperature resulting in a crude methanol extract (156.11 g). After the dissolution of the crude methanol extract in MeOH:EtOAc (1:1, 6 L) and solvent removal, the crude MeOH:EtOAc (1:1) soluble fraction (81.11 g) was obtained. The crude of 1:1 MeOH:EtOAc was separated by Si-gel CC (SiO_2_, 500 g, CH_2_Cl_2_-hexane and MeOH-CH_2_Cl_2_ gradients) which gave rise to fractions A1 to A7. Fraction A3 (3.20 g) produced fractions B1 to B5 after Si-gel CC (10% acetone-hexane isocratic). The B4 fraction yielded white needle-shaped crystals after recrystallization from MeOH-CH_2_Cl_2_ and was identified as betulinic acid or BA (130.11 mg) (Figure 1) (Figure S1, see Supplementary Material). The BA was verified by comparing the physical properties and spectroscopic data with those reported in the literature [14]. The BA was initially diluted in DMSO at a stock solution of 1 mg/mL and stored at −20 °C inside a dark tube for further use.

### 4.4. Cell Lines and Cell Culture

Human colorectal carcinoma cell lines: SW480 (ATCC CCL-228) and HCT116 (ATCC CCL-247) were purchased from the American Type Culture Collections (ATCC, Manassas, VA, USA). Cells were cultured in different media including Dulbecco’ s modified Eagle’ s medium (DMEM) and RPMI 1640 (Gibco, Grand Island, NY, USA) containing 10% fetal calf serum and 1% penicillin/streptomycin (Gibco, Grand Island, NY, USA). Cells were incubated in a humidified incubator with 5% CO2 at 37 °C.

### 4.5. Cell Viability Assays

The cell cytotoxicity or cell viability of BA treated human colorectal cancer cells was performed by MTT assay. In brief, HCT116 and SW480 cells were grown in 96-well culture plates at the density of 8 × 10^3^ cells per well for 24 h. Both cells were treated with ranging concentrations of BA (1.5625, 3.125, 6.25, 12.5, 25, 50, 75, 100 µg/mL) for 24 and 48 h. After incubation for 24 and 48 h, 10 µL of 5 mg/mL MTT was added to each well, followed by incubation at 37 °C for 3 h. Then, the medium was removed and replaced with an isopropanol solution to dissolve the formazan crystals. Viable cells were detected by measuring the absorbance at 562 nm using a microplate reader (ELx808TM, BioTek Instruments, Inc., Winooski, VT, USA). The experiments were repeated three times independently.

### 4.6. Cell and Nucleus Morphological Analysis

The morphology of cells and nuclei were assessed after treatment with BA in human colorectal cancer cells by light microscopy. Both cancer cell lines were seeded onto the sterile coverslips which were placed onto the bottom of each well of a 6-well plate, and maintained at 37 °C, overnight. Then, cells were treated with BA in ranging concentrations (0–10 μM). After 24 h, cells were washed with PBS (GIBCO, Grand Island, NY, USA) and fixed in 4% paraformaldehyde (Sigma-Aldrich, St. Louis, MO, USA) for 15 min. Then, the final concentration of 5 µg/mL of Hoechst 33342 nuclear staining was added to each well followed by incubation for 30 min. Finally, the coverslips were placed onto clean glass slides and mounted with a 10% glycerol buffer. Morphology of the cells was visualized under a phase contrast microscope. Concurrently, apoptosis changes in nuclear morphology were observed under a fluorescence microscope (Olympus BX53 Digital Fluorescence Microscope, Olympus, Chiba, Japan). Three coverslips were used for each experimental group. The experiments were performed in triplicate, independently.

### 4.7. Cell Apoptotic Analysis

Apoptotic cells were verified by the Annexin V-FITC Apoptosis Detection Kit according to the manufacturer’s instructions. Flow cytometry was used to analyze apoptosis between the control and the BA-treated cells at 24 h post treatment. Subsequently, HCT116 and SW480 cells were incubated with BA at the indicated concentrations (i.e., 0–5 µM in HCT116 and 0–10 µM in SW480). Cells were harvested and washed with trypsin/EDTA followed by a single wash with ice-cold PBS. Next, the cells were stained with 5 μL of annexin V-FITC and 5 μL of propidium iodide (PI), then incubated for 15 min at room temperature. The final working volume was adjusted by adding 400 μL of binding buffer before the flow cytometry analysis was performed. Finally, cells were collected and analyzed by counting normal cells, early apoptotic cells, and late apoptotic/necrotic cells in three areas in the microscope’s field of view. All experiments were carried out independently in triplicate.

### 4.8. Western Blot Analysis

The effect of BA on molecular signaling in HCT116 and SW480 were evaluated by the western blot analysis. In brief, both cancer cells were treated with BA at the indicated concentrations. The cells were washed with ice-cold PBS, and protein extraction was performed using RIPA buffer (Cell Signaling Technology, Danvers, MA, USA). Total protein concentration was determined using the Bicinchoninic Acid Protein Assay Kit (Thermo Fisher Scientific, Waltham, MA, USA). The protein samples were separated by sodium dodecyl sulphate polyacrylamide gel electrophoresis (SDS-PAGE) followed by a sequential transfer to the nitrocellulose membrane. To prevent non-specific protein binding, the membrane was blocked with 5% skim milk for 1 h at room temperature, before incubating with primary antibodies overnight at 4 °C. Then, the membrane was incubated in a secondary antibody with agitation for 2 h at room temperature. Immunoreactive bands were detected with the enhanced chemiluminescence system (Thermo Fisher Scientific, Waltham, MA, USA). Triplicate experiments were carried out.

### 4.9. Molecular Docking

To predict whether the interaction between BA and HSPA is a direct interaction or not, molecular docking was performed in PyMOL (v2.1 INTEL-12.10.16). We chose the HSPA complex belonging to *Homo sapiens sp* HSP70 (PDB ID 5AQF), which was also investigated by Williamson et al. [51] for studying the binding between HSPA and adenosine-derived inhibitors which would be used for comparison to the present study. Coordinate and topology files for the NBD were prepared using the AutoDockTools (v1.5.6). Water molecules were removed, and implicit hydrogens were added followed by addition of Kollman charges. Subsequently, BA and ADP were docked using the PyRx virtual screening tool.

### 4.10. Statistical Analysis

GraphPad Prism version 8.0 software (GraphPad Software, Inc., La Jolla, CA, USA) was used for the entire statistical analysis. All data were expressed as mean ± SEM and all experiments were repeated three times. Comparison between control and BA treatments were performed using one-way ANOVA with Dunnett’s post hoc test. The level of significance was considered significant (*) when *p* < 0.05, more significant (**) when *p* < 0.01, and very significant (***) when *p* < 0.001.

## 5. Conclusions

In conclusion, our results indicated that BA can modulate the expression of HSPA and induce an intrinsic apoptosis pathway in HCT116 and SW480 CRC cells. This finding contributes to the current understanding of BA in CRC and its potential interaction with HSPA. Although further studies are needed understand the BA-HSPA interaction, our findings indicate that BA can be developed as a potential therapeutic in the treatment of CRC.

## Data Availability

The data supporting the conclusion in this study are available on request from the corresponding author.

## Supplementary Materials

The following are available online, Figure S1: NMR spectra of betulinic acid; A ^1^H-NMR spectrum (500 MHz, CD_2_Cl_2_), B ^13^C-NMR spectrum (125 MHz, CD_2_Cl_2_).

## Sample Availability

Samples of the compounds are not available from the authors.
