# Supplementary material for: Betulinic Acid Modulates the Expression of HSPA and Activates Apoptosis in Two Cell Lines of Human Colorectal Cancer
Source: Molecules. 2021 Oct 22;26(21):6377. doi: 10.3390/molecules26216377 (PMC8588033; doi:10.3390/molecules26216377)
Supplement: Supplementary file 1 [file molecules-26-06377-s001.zip › molecules-1225982-supplementary.pdf]

**Supplementary Result for BA**

Betulinic acid (BA) extraction from leaves and twigs of *Piper retrofractum* was obtained as white crystalline from methanol-dichloromethane, m.p. 278-279 °C (Lit 282 °C); FT-IR (KBr) $\upsilon_{max}$ = 3451 (O-H stretching), 2942, 1639 (C=O stretching), 1453, 1376, 1299, 1047, 1022, 959, 881EIMS m/z (rel. int.): 465 (4), 411 (25), 381 (8), 298 (39), 219 (15), 203 (55), 189 (100); optical rotation: $[\alpha]_{589}^{30}$ +8° (*c* 0.1, pyridine).

BA was obtained as white crystalline compound with dichloromethane in methanol, m.p. 278-279 °C. Its EIMS showed [M]+ peak at m/z 456 which established the molecular formula of $C_{30}H_{48}O_3$. The FTIR spectrum showed OH-stretching band at 3451 $cm^{-1}$ and C=O stretching of carboxylic acid at 1639 $cm^{-1}$. The 1H NMR (500 MHz, p) spectrum showed the presence of methine proton on the carbon connected to oxygen in the structure was indicated by the multiplet at δ 3.50 (1H, t, H-3), two olefinic protons at δ 4.84 (1H (s)) and δ 4.95 (1H (s)). The signal at 0.80 (H-25), 1.10 (H-24), 1.14 (H-26), 1.20 (H-27) and 1.20 (H-23). This compound was proved to possess an isopropenyl group by the signals of a vinyl methyl at δ 1.78 (s), and the olefinic methylene protons at δ 4.80 (brs, H-29a) and 4.95 (brs, H-29b). The 125 MHz 13C-NMR spectrum of this compound exhibited thirty signals for thirty carbons. The DEPT spectra indicated the present of the present of six methyl carbons, ten methylene carbons, six methine carbons, six quaternary carbons and one carbonyl carbon

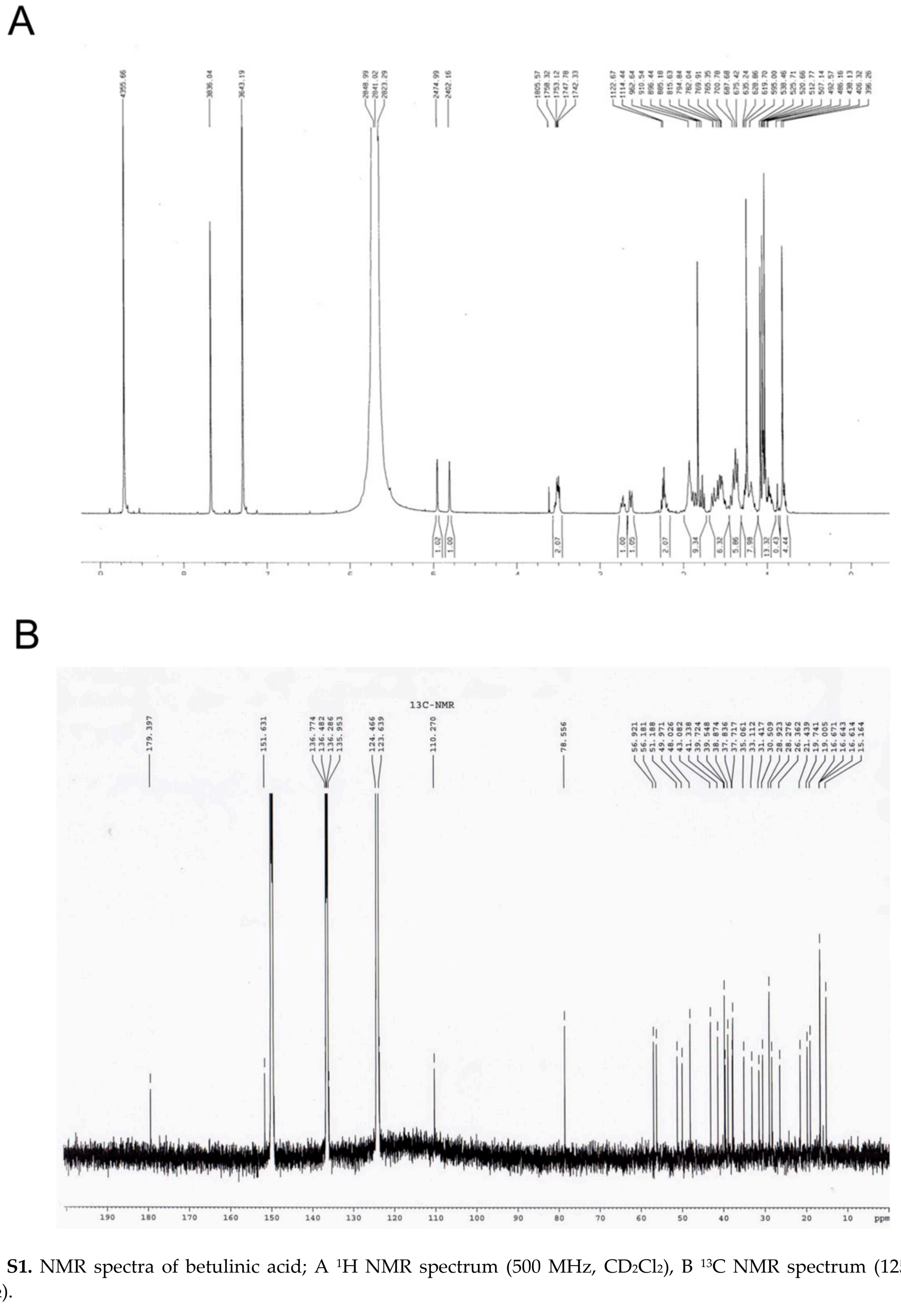


**Figure S1.** NMR spectra of betulinic acid; A $^{1}$H NMR spectrum (500 MHz, $CD_2Cl_2$), B $^{13}$C NMR spectrum (125 MHz, $CD_2Cl_2$).
